# Accelerating the Design of Double-Absorber Solar Cells: From Surrogate Model-Assisted Reinforcement Learning and Multi-Algorithm Optimization Comparison to Transfer Learning

**DOI:** 10.3390/ma19143091

**Published:** 2026-07-17

**Authors:** Yuhan Zhang, Qiaochu Sun, Jiang Zhao

**Affiliations:** College of Integrated Circuit Science and Engineering, Nanjing University of Posts and Telecommunications, Nanjing 210023, China; b23030218@njupt.edu.cn (Y.Z.); b24030210@njupt.edu.cn (Q.S.)

**Keywords:** lead-free perovskite solar cells, double-absorber solar cells, Cs_2_TiBr_6_/RbGeI_3_, SCAPS-1D, surrogate model, reinforcement learning, transfer learning

## Abstract

Lead-free double-absorber perovskite solar cells offer broad-spectrum absorption and environmental benefits, but their multilayer heterostructure creates computational challenges for conventional design optimization. This study introduces an automated framework integrating SCAPS-1D simulation, multilayer perceptron (MLP) surrogate modeling, metaheuristic algorithms, and reinforcement learning (RL). Using FTO/ZnO/Cs_2_TiBr_6_/RbGeI_3_/CuI/Au cells, the MLP model trained on Latin hypercube sampling data achieved high accuracy (R^2^ > 0.95). The proximal policy optimization (PPO) RL agent converged to 27.41% power conversion efficiency (PCE) in approximately 20 steps. For direct 15-dimensional optimization, simulated annealing and particle swarm optimization reached 98% target PCE with 138 and 111 function evaluations, respectively, while Grey Wolf Optimizer (GWO) yielded the highest average PCE. Transfer learning successfully adapted the pretrained model to a novel FASnI_3_/Sb_2_S_3_ structure, improving the prediction accuracy of PCE, J_SC_, and FF. This work systematically optimizes Cs_2_TiBr_6_/RbGeI_3_ solar cells while establishing an efficient, generalizable paradigm for intelligent photovoltaic device design, validation, and material discovery.

## 1. Introduction

Against the grand historical backdrop of the global energy structure’s transition towards zero-carbon emissions, solar energy has received widespread attention as a viable renewable energy source [1]. Although silicon-based photovoltaic technology still dominates the market, perovskite solar cells (PSCs) exhibit immense commercialization potential due to their exceptional light absorption coefficients, extremely long carrier diffusion lengths, low manufacturing costs, and highly flexible solution-processing techniques [2,3]. However, the environmental and health concerns associated with lead-based perovskites have hindered their further application and development, highlighting the urgent need to develop lead-free perovskite materials and device architectures. Therefore, the focus of this study lies on two lead-free perovskite materials: Cs_2_TiBr_6_ and RbGeI_3_. Cs_2_TiBr_6_ belongs to the A_2_BX_6_-type vacancy-ordered double perovskites [4]. Due to its direct bandgap characteristics, it can efficiently absorb visible light [5]. Studies have shown that through DFT calculations and SCAPS-1D simulations, solar cell structures using Cs_2_TiBr_6_ as the absorber layer have achieved a PCE of 24.82% [6]. Furthermore, Cs_2_TiBr_6_ exhibits excellent stability [7] and maintains dynamic stability even under applied pressure [8]. RbGeI_3_ is a germanium-based lead-free halide perovskite with strong light absorption and carrier transport capabilities [9]. Research indicates that solar cell structures employing RbGeI_3_ as the absorber layer have reached a PCE of 24.03% [10].

The double-absorber layer device configuration has emerged as a crucial approach to enhancing PSC performance [11]. By integrating absorber layers with different bandgaps, the solar spectrum can be utilized more effectively, thereby increasing the short-circuit current [12]. Numerous studies have demonstrated that double-absorber devices can achieve a PCE of over 30% [13,14]. Additionally, research has confirmed that the efficiency of perovskite/silicon tandem solar cells has reached 32.5% [15] and continues to advance toward even higher efficiencies. Despite the significant advantages of the double-absorber structure, it inherently introduces complexity into device design. This multilayer architecture involves numerous independent physical parameters, forming a high-dimensional and strongly coupled parameter space [16]. Traditional trial-and-error methods based on SCAPS-1D suffer from poor search efficiency and limited coverage in high-dimensional parameter spaces, while global optimization requires massive computational efforts, resulting in a severe computational cost bottleneck [17].

To address this challenge, the application of machine learning (ML) in photovoltaic device design has increasingly garnered attention, aiming to accelerate material discovery, optimize devices, and guide experiments [18,19,20]. Currently, ML applications in PSCs primarily focus on constructing surrogate models to accelerate simulation predictions. These surrogate models can learn the input-output relationships of SCAPS-1D simulation results, significantly reducing computational costs to enable millisecond-level performance predictions. For instance, by employing models such as Support Vector Regression (SVR), Random Forest (RF), and eXtreme Gradient Boosting (XGBoost), the efficiency of Sb_2_S_3_/Sb_2_Se_3_ double-absorber solar cells can be optimized [21]. In the optimization of Rb_2_ScCuCl_6_-based perovskite solar cells, Artificial Neural Network (ANN) models were trained to predict the performance impact of key parameters, achieving a certified efficiency of over 26% [22]. Beyond constructing surrogate models, some studies have directly coupled intelligent optimization algorithms with SCAPS-1D, which has also proven effective in enhancing solar cell performance. Particle Swarm Optimization (PSO) and Simulated Annealing (SA) have been utilized to optimize the multilayer thicknesses of solar cells, leading to a significant reduction in the number of function evaluations compared to brute-force methods [23]. Genetic Algorithms (GAs) have been used to optimize four-dimensional parameters, including defect density and thickness, yielding optimal parameter combinations [24]. A penalty-based Differential Evolution (P-DE) algorithm was employed to optimally extract hidden electrical parameters in the double-diode model of solar photovoltaic modules, demonstrating that the algorithm significantly outperforms traditional methods in both convergence speed and fitting accuracy [25]. However, current research still exhibits gaps and deficiencies regarding multi-algorithm benchmark comparisons, reinforcement learning, and cross-structural generalization. Most ML studies focus on static surrogate models and Bayesian optimization (BO) [26], while the potential of reinforcement learning, particularly its capacity for adaptive optimization in continuous action spaces and its strategic characteristics has not yet been fully validated in photovoltaic device design. Concurrently, when facing data scarcity for novel devices of similar structures, traditional ML methods require substantial amounts of data to train reliable models, thereby limiting their rapid application. Transfer learning, as a strategy that leverages existing model knowledge to improve learning on new tasks, provides an effective pathway to overcome this data scarcity problem [27,28].

This study proposes a full-pipeline automated optimization framework aimed at accelerating the design of double-absorber solar cells. This framework encompasses data-driven surrogate model construction, multi-dimensional automated optimization and comparative analysis (combining traditional heuristic algorithms, BO, and reinforcement learning), as well as transfer learning to verify the high generalization capability of the models. Specifically, a physical model of the perovskite solar cell is first constructed using SCAPS-1D simulation software, and a Latin Hypercube Sampling (LHS) method is employed to efficiently collect data for training a high-precision Multilayer Perceptron (MLP) surrogate model. Secondly, automated operation of SCAPS-1D is achieved via Python scripts, enabling the optimization algorithms to control the physical simulations. Building on this foundation, this paper comparatively evaluates the optimization performance of meta-heuristic algorithms, including GA [29], PSO [30], Differential Evolution (DE) [31], SA [32], and Grey Wolf Optimizer (GWO) [33], as well as BO [34] and a PPO-based RL agent [35], under both the surrogate model and the SCAPS-1D physical simulation environments. Finally, by applying the pre-trained surrogate model to a novel lead-free double-absorber structure, the effectiveness of transfer learning in enhancing model generalization performance under small sample sizes is verified, providing a new paradigm for the intelligent design and material discovery of future photovoltaic devices.

## 2. Methods

**Figure 1:**
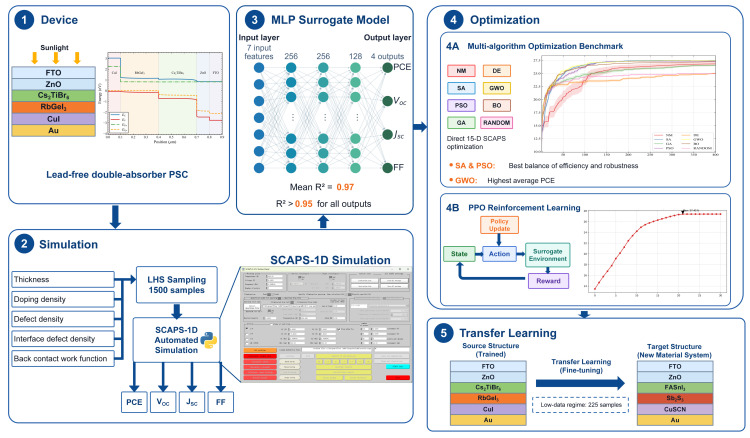
Overall workflow of the proposed automated optimization framework.

The overall workflow of the proposed automated optimization framework is illustrated in Figure 1, including device construction, automated SCAPS-1D simulation, MLP surrogate modeling, multi-algorithm optimization, PPO-based reinforcement learning, and transfer learning.

### 2.1. Device Physics Models and Data Acquisition

#### 2.1.1. SCAPS-1D Simulation Software

This study selected the SCAPS-1D numerical simulation software developed by Ghent University in Belgium [36] to simulate the performance of one-dimensional solar cells. This software models the behavior of solar cells by solving the Poisson equation, the electron continuity equation, and the hole continuity equation.

#### 2.1.2. Device Structure Definition

To achieve maximum absorption efficiency, the wider-bandgap layer among the absorber layers must be designed on the light-facing side to absorb shorter-wavelength light and transmit longer-wavelength light. Conversely, the narrower-bandgap layer should be placed on the rear side to absorb the longer-wavelength light. Based on this rationale, this study designed a device structure of FTO/ETL/Cs_2_TiBr_6_/RbGeI_3_/HTL/Au. Subsequently, the Hole Transport Layer (HTL) and Electron Transport Layer (ETL) were selected according to the principle of energy level matching. To ensure that holes are efficiently extracted into the hole transport layer, the valence band offset (VBO) at the RbGeI_3_/HTL interface should be close to 0. To block electrons and avoid recombination losses, the conduction band offset (CBO) at the RbGeI_3_/HTL interface should be a large positive value. A similar logic applies to the ETL/Cs_2_TiBr_6_ interface. The energy level comparison diagrams for 10 different HTLs and 10 different ETLs are provided in Supplementary Information Figures S1 and S2. Ultimately, ZnO was selected as the electron transport layer and CuI as the hole transport layer.

**Figure 2:**
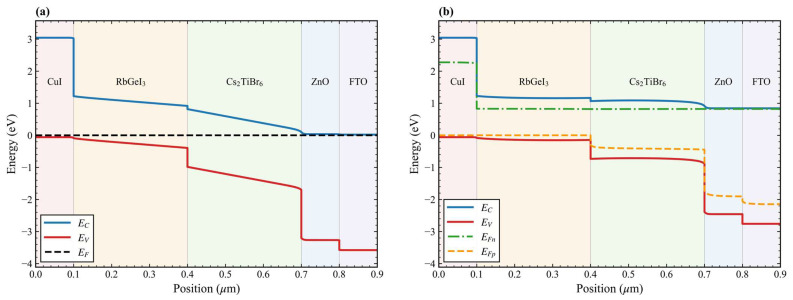
(**a**) Thermal-equilibrium band diagram. (**b**) Band diagram under light and open-circuit conditions.

The finalized device structure is FTO/ZnO/Cs_2_TiBr_6_/RbGeI_3_/CuI/Au. The SCAPS-1D software (Version number: 3.3.12) simulation input parameters are detailed in Table 1. The interface defect density between each layer was initially set to 1 × 10^14^ cm^−2^, resulting in a device power conversion efficiency (PCE) of 16.35%. Figure 2 presents the energy band diagrams of the device under two representative conditions. Figure 2a shows the thermal-equilibrium band diagram in the dark and without external bias. Figure 2b shows the band diagram under standard AM1.5G illumination at 300 K under open-circuit conditions.

LHS is an effective statistical sampling method. Compared to simple Monte Carlo random sampling, LHS can cover a high-dimensional parameter space with fewer sample points. In supervised learning tasks, larger datasets generally yield better results; however, to minimize training costs, the LHS method can be employed to achieve equivalent quality results using less data [42]. The dataset utilized in this study was derived from the SCAPS-1D simulation software. The input parameters were obtained by sampling the absorber layer thicknesses, doping concentrations, and defect densities within specific value ranges using the LHS method, as outlined in Table 2. The complete dataset generated from running the simulations is presented in Table 3.

### 2.2. Data-Driven Surrogate Model Construction and Training

Neural networks, originating from the mimicry of the human brain’s structure and learning mechanisms, are capable of approximating arbitrarily complex functions through nonlinear activation functions and multilayer architectures. In the domain of one-dimensional perovskite solar cell modeling, neural networks possess the capability to capture the complex nonlinear relationships between various parameters within the material layers and the resulting output performance. In this study, a multilayer perceptron (MLP) network was constructed to perform high-order nonlinear mapping on the input features. This network adopts a fully connected architecture, designed to extract complex data patterns through layer-by-layer propagation. The overall network architecture comprises one input layer, three hidden layers, and one output layer. The neuron counts for the hidden layers are configured sequentially as 256, 256, and 128. The input layer receives 7 distinct material layer parameter features, which, after dimensional mapping via a fully connected layer, are fed into a rectified linear unit (ReLU) activation function. Subsequently, a one-dimensional batch normalization layer is applied to standardize the local feature distribution during each iteration. This facilitates accelerated network convergence and enhances training stability. To improve the model’s generalization capability and prevent overfitting, the output of the batch normalization layer is then regularized through a Dropout layer with a dropout rate set to 10%. During each training iteration, the Dropout mechanism randomly sets the outputs of a fraction of the neurons to zero. After three rounds of the aforementioned hidden layer processing, the extracted 128-dimensional features are fed into the final linear output layer for mapping to the performance parameters: PCE, J_SC_, V_OC_, and FF.

Data preprocessing is crucial to the training outcomes and stability of neural networks. Therefore, prior to feeding data into the network, appropriate transformations and standardizations were applied to the feature space. Considering the significant range discrepancies among the physical variables, a base-10 logarithmic transformation was performed on the five density-related parameters within the input features. This nonlinear transformation drastically reduces the order-of-magnitude differences among the feature values, enabling smoother network processing of these exponentially varying physical quantities and laying the groundwork for subsequent optimization using the surrogate model. Following this, all input features and output targets were standardized.

To maximize the utilization of feature information within the limited development data and reduce random errors caused by a single data split, a 5-fold cross-validation strategy was adopted. The complete dataset was partitioned into training, validation, and test sets according to a ratio of 0.64:0.16:0.2, respectively. The test set was independently set aside prior to cross-validation. During each fold’s training, the remaining data was dynamically allocated into training and validation sets. Upon the completion of training, the model from the fold exhibiting the lowest validation loss was selected as the definitive model and subsequently evaluated on the test set.

During model training, Mean Squared Error (MSE) was selected as the loss function, and the Adam optimizer was employed with an initial learning rate of 1 × 10^−3^ and a weight decay of 1 × 10^−5^ to update the network weights. To further refine the training process, a dynamic learning rate scheduler based on validation loss was introduced. When the validation loss exhibited no improvement over 8 consecutive epochs, the learning rate automatically decayed by half, allowing the model to conduct finer convergence exploration near local minima.

To strictly prevent overfitting, an early stopping mechanism with a patience of 30 epochs was configured. During the training process, the validation loss was monitored in real-time. Once the model’s performance on the validation set ceased to improve for 30 consecutive epochs, training was forcibly terminated. The algorithm then automatically rolled back and saved the optimal model weights corresponding to the minimum validation loss achieved within that fold.

### 2.3. Design of the Automated Multidimensional Optimization Framework

At present, SCAPS-1D only supports numerical simulation with fixed, predefined material parameters. These parameters must be specified before the simulation starts and cannot be modified once the simulation is running. In solar-cell optimization, the next trial point is usually determined only after the completed simulation results have been obtained and analyzed. To enable different optimization algorithms to directly invoke SCAPS-1D and obtain accurate numerical solutions, an automated optimization framework was first established to programmatically update the simulation inputs and retrieve the outputs. In this study, Python (Version number: 3.12.3) scripts were used to execute SCAPS-1D preset files, while the SCAPS-1D scripting interface was employed to collect and update the simulation results. During each simulation cycle, the input parameters were updated by dynamically parsing and rewriting the underlying SCAPS-1D definition files (.scaps) and material property files (.material). The resulting simulation outputs were then stored in memory, thereby enabling the construction of an automated simulation framework. This framework serves as a bridge between the underlying physical model and the higher-level optimization algorithms, enabling continuous and unattended iterative optimization of the target device. A representative demonstration of this automated optimization process is provided in Supplementary Video S1.

Based on this automated simulation framework, several optimization methods were investigated, including derivative-free optimization, metaheuristic algorithms, Bayesian Optimization (BO), and reinforcement learning.

In machine learning and optimization, metaheuristic algorithms are widely used to solve complex global optimization problems, especially when the objective function is non-convex, non-differentiable, or associated with a high-dimensional search space. The metaheuristic algorithms considered in this study include the Genetic Algorithm (GA), Particle Swarm Optimization (PSO), Differential Evolution (DE), Simulated Annealing (SA), and Grey Wolf Optimizer (GWO). These algorithms are inspired by natural optimization mechanisms and iteratively search for near-optimal solutions. GA generates the next-generation offspring $x_{i}^{\left(t+1\right)}$ through a linear combination of selected parent solutions combined with a perturbation vector, while applying a boundary operator B(·) to ensure compliance with predefined physical constraints, as shown in Equation (1).

(1) $$x_{i}^{\left(t+1\right)}=B[\lambda_{i}x_{p_{1}}^{\left(t\right)}+(1-\lambda_{i})x_{p_{2}}^{\left(t\right)}+\sigma_{t}\varepsilon_{i}]$$

PSO updates the velocity $v_{i}^{\left(t+1\right)}$ and position $x_{i}^{\left(t+1\right)}$ of each particle by guiding them toward their personal best position $p_{i}^{\left(t\right)}$ and the global best position $g^{\left(t\right)}$ while enforcing boundary constraints, as shown in Equations (2) and (3).

(2) $$v_{i}^{\left(t+1\right)}=\omega v_{i}^{\left(t\right)}+c_{1}r_{1}(p_{i}^{\left(t\right)}-x_{i}^{\left(t\right)})+c_{2}r_{2}(g^{\left(t\right)}-x_{i}^{\left(t\right)})$$

(3) $$x_{i}^{\left(t+1\right)}=B[x_{i}^{\left(t\right)}+v_{i}^{\left(t+1\right)}]$$

DE constructs a mutant vector $m_{i}^{\left(t\right)}$ by adding a scaled difference between two random population vectors to a third vector, which serves as the foundation for subsequent crossover and greedy selection steps, as shown in Equation (4).

(4) $$m_{i}^{\left(t\right)}=x_{r_{1}}^{\left(t\right)}+F(x_{r_{2}}^{\left(t\right)}-x_{r_{3}}^{\left(t\right)})$$

SA produces a perturbed candidate solution and accepts inferior solutions with a temperature-dependent probability $e^{\left(\Delta f/T_{t}\right)}$, allowing the algorithm to escape local optima during early high-temperature stages.

GWO updates each candidate solution $X_{i}^{\left(t+1\right)}$ by taking the arithmetic average of the three best solutions (α, β, and δ), followed by a boundary-handling operation to restrict parameters within their physical limits.

(5) $$X_{i}^{\left(t+1\right)}=B[(X_{1}+X_{2}+X_{3})/3]$$

BO constructs a probabilistic surrogate model of the objective function and, based on the historical observation set D_t_, iteratively selects the next parameter set x_t + 1_by maximizing the acquisition function α(x), as shown in Equation (6).

(6) $$x_{t+1}={\mathrm{argmax}}_{x}\alpha (x|D_{t})$$

RL centers on an agent that interacts with an environment and learns an optimal policy through trial and error to maximize cumulative reward. In this study, an agent based on the PPO algorithm was constructed for efficient and adaptive parameter optimization on the pretrained surrogate model. The RL environment was built on the previously trained MLP surrogate model. The automated physical simulation framework was not used for RL training because RL typically requires hundreds of thousands of trial-and-error interactions, making direct device-level simulation prohibitively expensive compared with the surrogate model. The state space of the RL agent was defined as a seven-dimensional feature vector after logarithmic transformation and standardization, including the thicknesses, defect densities, and doping concentrations of the two absorber layers. To ensure that the exploration range remained physically meaningful and stayed within the region covered by the surrogate-model training data, the state space was strictly constrained within the distribution of the MLP training samples. The action space was defined as the fine-tuning step size applied to the current state vector. To enable precise local search, the single-step action magnitude was restricted to the continuous normalized interval [−0.15, 0.15].

In the RL framework, the reward function guides the agent’s learning process and shapes its optimization behavior. A piecewise reward function was designed based on differential reward shaping. At time step t, the predicted power conversion efficiency of the current state is denoted by η_t_, and the efficiency increment relative to the previous step is defined as $\Delta \eta_{t}\;=\eta_{t}\;-\eta_{t-1}$. The mathematical expression of the reward function Rt is given in Equation (7).

(7) $$R_{t}=\left\{\begin{array}{ll} -10.0, & \eta_{t}<0\;\mathrm{or}\;\eta_{t}>35\\ 0.05\eta_{t}+2.0\Delta \eta_{t}, & 0\leq \eta_{t}\leq 35\;\mathrm{and}\;\Delta \eta_{t}>0\\ 0.015\eta_{t}+2.0\Delta \eta_{t}, & 0\leq \eta_{t}\leq 35\;\mathrm{and}\;\Delta \eta_{t}\leq 0 \end{array}\right.$$

If the agent explores a state that causes the surrogate model output to fall outside the physically reasonable range, a severe negative reward of −10 is immediately assigned and the current episode is terminated. For physically valid states, the reward consists of two parts: a baseline reward determined by the absolute PCE value and a differential reward determined by the change in PCE. When the PCE increases, the agent receives an amplified positive reward.

### 2.4. Transfer Learning-Based Generalization Strategy

Under traditional machine learning paradigms, sufficient labeled data is required to train reliable models. However, in practical material discovery and device optimization, newly proposed solar cell structures often lack adequate simulation and experimental data for model training. To rapidly construct high-precision surrogate models for new solar cell structures under conditions of limited data, transfer learning is introduced in this study. Transfer learning is a modeling scheme that facilitates knowledge transfer, capable of leveraging knowledge from a source domain to improve learning tasks in a target domain. This approach is particularly effective when there is an asymmetry in the volume of knowledge between the source and target domains. This efficacy stems from the pre-trained model’s ability to learn universal physical mapping rules and high-order feature representations from the massive dataset in the source domain, thereby assisting the target domain model in converging rapidly, an outcome difficult to achieve by directly training a model solely on the small dataset of the target domain.

Transfer learning based on parameter fine-tuning requires the inputs and outputs of both the source and target domains to remain consistent. The trained surrogate model was utilized as the pre-trained model, and a perovskite solar cell with an FTO/ZnO/FASnI_3_/Sb_2_S_3_/CuSCN structure was employed as the target domain. This structure also features a double-absorber architecture, and its analysis and modeling have been detailed in previous work. For the target domain structure, the LHS method was utilized to sample the absorber layer thicknesses, doping concentrations, and defect densities within the same value ranges as those in the source domain. Simulations yielded 1500 sets of data, which were subsequently divided randomly into two groups: 15% of the data (225 samples) served as the fine-tuning training set, while 85% of the data (1275 samples) constituted the independent test set.

During the data preprocessing stage, the identical base-10 logarithmic transformation was first applied to the density-related parameters within the target domain data. To guarantee feature space alignment and mitigate the effects of input feature shifts, the standard scaler generated during the source domain pre-training phase was directly frozen and invoked to transform the input features and output targets of the target domain. In the initialization phase of the transfer learning network, the model directly loaded the MLP weights that achieved optimal performance in the source domain. During the fine-tuning training process, all neuron weights were permitted to update. Concurrently, the fine-tuning process continued to employ the 5-fold cross-validation, dynamic learning rate decay, and early stopping mechanisms to select the optimal model.

To evaluate the effectiveness and possible limitations of transfer learning, a comparative experiment trained entirely from scratch was designed using the identical fine-tuning training set. The predictive performances of the directly trained model and the transfer-learning model were evaluated on the same independent test set. For each output k, the root mean square error (RMSE), mean absolute error (MAE), coefficient of determination R^2^, and normalized RMSE (NRMSE) were calculated as Equations (8)–(11).

(8) $${\mathrm{RMSE}}_{k}=\sqrt{\frac{1}{n}\sum_{i=1}^{n}{\left({\hat{y}}_{i,k}-y_{i,k}\right)}^{2}}$$

(9) $${\mathrm{MAE}}_{k}=\frac{1}{n}\sum_{i=1}^{n}\left|{\hat{y}}_{i,k}-y_{i,k}\right|$$

(10) $$R_{k}^{2}=1-\frac{\sum_{i=1}^{n}{\left(y_{i,k}-{\hat{y}}_{i,k}\right)}^{2}}{\sum_{i=1}^{n}{\left(y_{i,k}-{\bar{y}}_{k}\right)}^{2}}$$

(11) $${\mathrm{NRMSE}}_{k}=\frac{{\mathrm{RMSE}}_{k}}{y_{k,\max}-y_{k,\min}}\times 100\%$$

where y_i,k_ and ŷ_i,k_ denote the true and predicted values of the k-th photovoltaic output for the i-th test sample, respectively; ȳ_k_ is the mean true value of the k-th output in the test set; y_k,max_ and y_k,min_ are the maximum and minimum true values of the corresponding output in the test set; and n is the number of test samples. RMSE and MAE retain the physical units of the corresponding output, whereas NRMSE provides a dimensionless metric for comparing the relative prediction errors among outputs with different scales. The model accuracy in the transfer-learning analysis was assessed using output-specific R^2^, RMSE, MAE, and NRMSE values. 

## 3. Results and Discussion

### 3.1. Performance Validation of the MLP Surrogate Model

**Figure 3:**
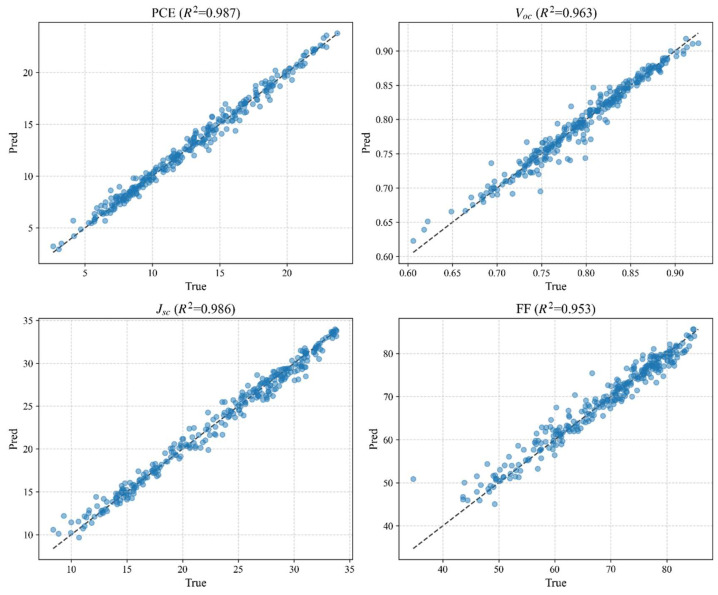
Scatter plots of the model’s predictions for PCE, V_OC_, J_SC_, and FF on the test set.

Based on the data presented in Table 3, an MLP neural network surrogate model is trained. The inputs to the model consist of the thicknesses of Cs_2_TiBr_6_ and RbGeI_3_, the doping concentrations of Cs_2_TiBr_6_ and RbGeI_3_, and the interface defect densities associated with Cs_2_TiBr_6_ and RbGeI_3_. The outputs of the model are the PCE, Open-Circuit Voltage (V_OC_), Short-Circuit Current Density (J_SC_), and Fill Factor (FF). Upon the completion of training, the performance of the optimal MLP surrogate model on the test set is illustrated in Figure 3. For the four performance parameters, the R^2^ values all exceeded 0.95. The four output heads of the model achieve an average MSE of 1.51, an average Mean Absolute Error (MAE) of 0.64, and an average coefficient of determination (R^2^) of 0.97. This demonstrates that the model can reliably replace SCAPS-1D for physical predictions with exceptionally high accuracy, particularly for the PCE and Jsc parameters.

### 3.2. Comprehensive Evaluation and Comparison of Automated Optimization Algorithms

Under the current mainstream research paradigm, machine learning is typically utilized to establish high-precision predictive models, followed by SHAP (SHapley Additive exPlanations) analysis to investigate feature importance. This approach guides the prioritization of subsequent laboratory processes, such as physical film preparation and passivation techniques. Given the premise that a reliable surrogate model is available to provide rapid mapping, Section 3.2.1 investigates the optimization performance of traditional derivative-free methods, various meta-heuristic algorithms, BO, and a PPO-based RL agent in conducting adaptive search for the optimal solution within the identical surrogate model environment. This section aims to eliminate the interference caused by the time-consuming underlying physical simulations, focusing on comparing the global search capabilities, convergence limits, and ultimate optimization accuracies of the various algorithms within a high-dimensional nonlinear space.

When confronted with novel device architectures lacking sufficient data to train surrogate models, researchers often must rely directly on physical simulation software. Therefore, Section 3.2.2 will integrate the SCAPS-1D automated optimization framework developed in Section 2.3 to directly drive the underlying physical model for iterative optimization using the aforementioned algorithms. This section will primarily investigate the trade-off relationship between optimization efficiency and optimization accuracy for each algorithm under the physical simulation environment.

#### 3.2.1. Comparison of Algorithm Optimization Accuracy Based on High-Precision Surrogate Models

Tests reveal significant differences in the optimization efficiency of various algorithms. However, due to the use of a surrogate model, the execution speed was highly accelerated, resulting in minimal differences in computational time among the different algorithms. To maximize the performance of each algorithm, a uniform limit on the number of iterations was not imposed; instead, an automatic stopping mechanism was adopted. For SA, PSO, GA, DE, GWO, and BO, optimization was automatically terminated when the number of function evaluations (NFE) exceeded 500 consecutive times without any performance improvement. The Nelder-Mead (NM) algorithm automatically stopped when the difference in objective function values or coordinates corresponding to each vertex of the simplex was less than or equal to 0.0001.

For the training process of the RL model, the total number of training iterations is set to 100,000. During the training process, the state space is reinitialized every 30 iterations (by randomly sampling points within the value range). During testing, the RL agent is allowed to optimize for 30 steps, and the highest PCE is taken as the final optimization result.

The optimization results obtained using different methods on the surrogate model are presented in Table 4. The test results indicate that BO exhibits the slowest convergence speed and a final PCE stagnating at 27.02%, representing the poorest optimization performance. SA, PSO, GA, GWO, and RL demonstrate the best optimization performance, reaching a maximum PCE of 27.41%.

**Figure 4:**
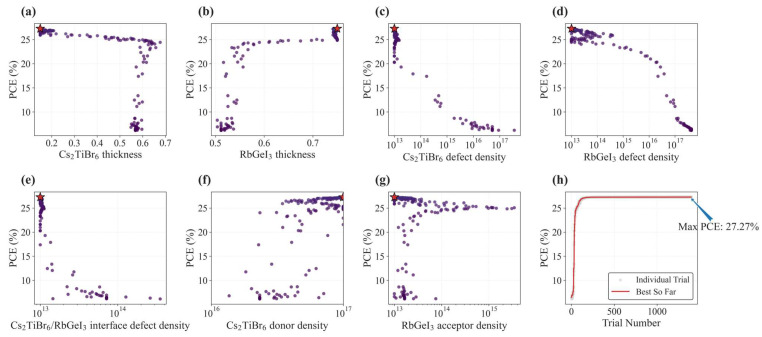
(**a**–**g**) Scatter plots of optimization parameters and (**h**) optimization history curve for the NM algorithm. In (**a**–**g**), the point color indicates the trial order, with lighter colors representing later trials, and the red stars mark the optimal solution. In (**h**), the gray dots represent individual trials, and the red line represents the best-so-far PCE.

**Figure 5:**
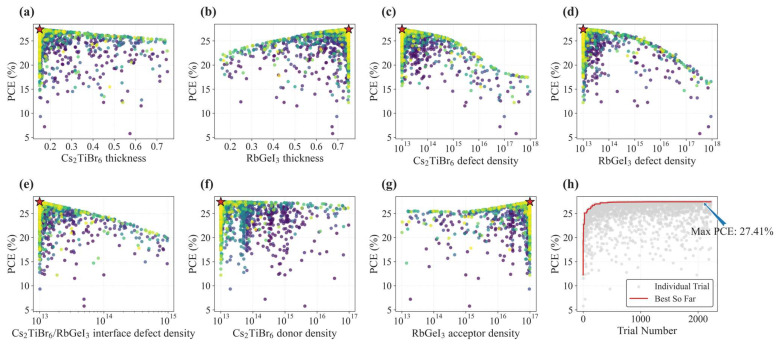
(**a**–**g**) Scatter plots of optimization parameters and (**h**) optimization history curve for the SA. In (**a**–**g**), the point color indicates the trial order, with lighter colors representing later trials, and the red stars mark the optimal solution. In (**h**), the gray dots represent individual trials, and the red line represents the best-so-far PCE.

**Figure 6:**
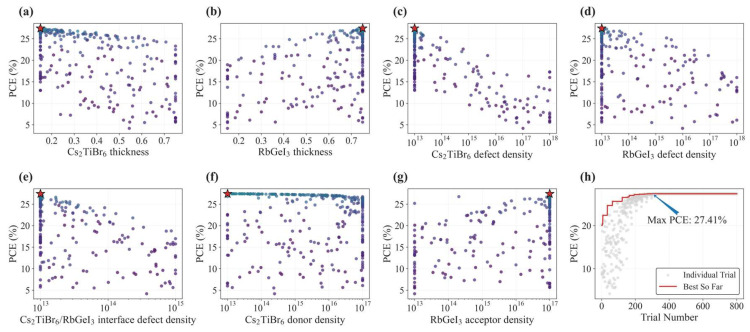
(**a**–**g**) Scatter plots of optimization parameters and (**h**) optimization history curve for the PSO algorithm. In (**a**–**g**), the point color indicates the trial order, with lighter colors representing later trials, and the red stars mark the optimal solution. In (**h**), the gray dots represent individual trials, and the red line represents the best-so-far PCE.

**Figure 7:**
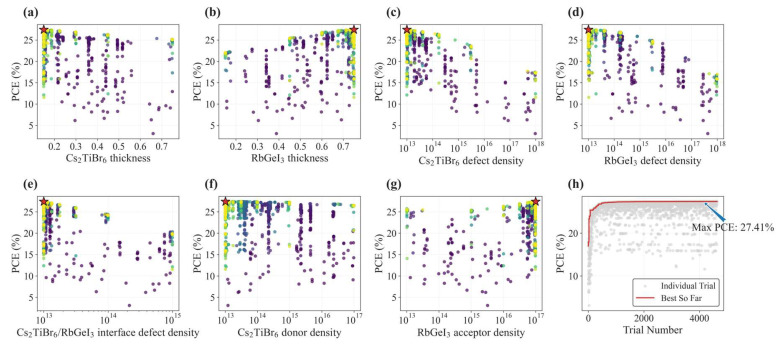
(**a**–**g**) Scatter plots of optimization parameters and (**h**) optimization history curve for the GA. In (**a**–**g**), the point color indicates the trial order, with lighter colors representing later trials, and the red stars mark the optimal solution. In (**h**), the gray dots represent individual trials, and the red line represents the best-so-far PCE.

**Figure 8:**
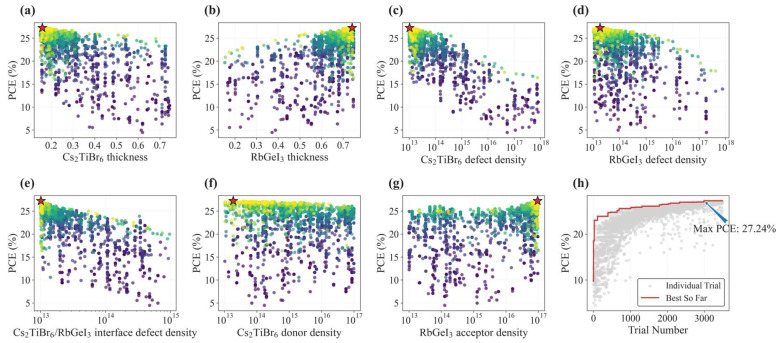
(**a**–**g**) Scatter plots of optimization parameters and (**h**) optimization history curve for the DE algorithm. In (**a**–**g**), the point color indicates the trial order, with lighter colors representing later trials, and the red stars mark the optimal solution. In (**h**), the gray dots represent individual trials, and the red line represents the best-so-far PCE.

**Figure 9:**
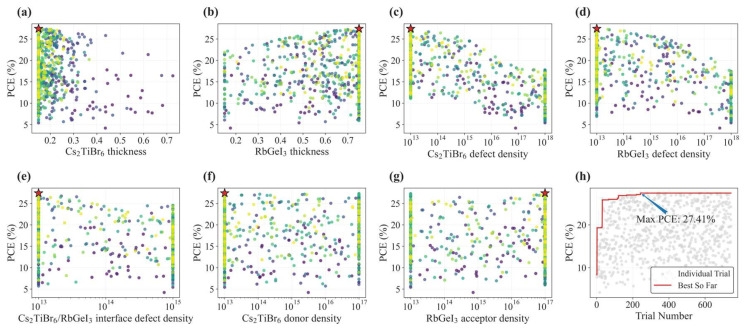
(**a**–**g**) Scatter plots of optimization parameters and (**h**) optimization history curve for the GWO algorithm. In (**a**–**g**), the point color indicates the trial order, with lighter colors representing later trials, and the red stars mark the optimal solution. In (**h**), the gray dots represent individual trials, and the red line represents the best-so-far PCE.

**Figure 10:**
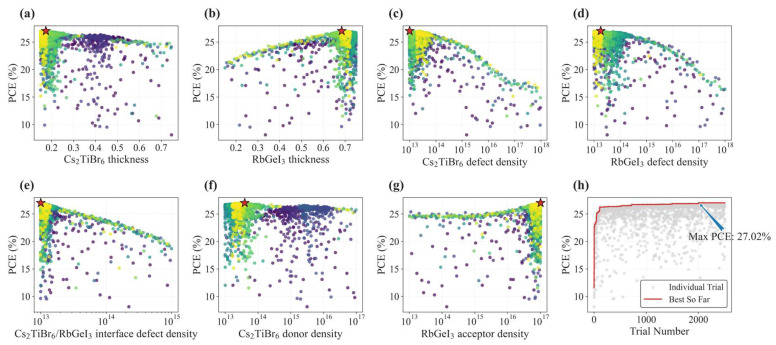
(**a**–**g**) Scatter plots of optimization parameters and (**h**) optimization history curve for the BO algorithm. In (**a**–**g**), the point color indicates the trial order, with lighter colors representing later trials, and the red stars mark the optimal solution. In (**h**), the gray dots represent individual trials, and the red line represents the best-so-far PCE.

Subfigures (a)–(g) in Figure 4, Figure 5, Figure 6, Figure 7, Figure 8, Figure 9 and Figure 10 present the optimization scatter plots of each algorithm concerning the thicknesses, defect densities, and doping concentrations of the double-absorber layers. A larger trial number corresponds to a lighter color, and the optimal values are marked with asterisks in the figures. Subfigure (h) in Figure 4, Figure 5, Figure 6, Figure 7, Figure 8, Figure 9 and Figure 10 illustrates the PCE optimization history of each algorithm, displaying the changes in PCE during each iteration and the overall optimization trend. For the NM algorithm, in Figure 4a–g, the number of scatter points is sparse, failing to form a widespread coverage network in the space. Instead, it presents a narrow trajectory path from a certain initial region to a local optimum. It is highly susceptible to the limitations of the initial point, easily falling into suboptimal local solutions. This inversely confirms the necessity of introducing a global optimization mechanism for the complex, high-dimensional parameter space of double-absorber perovskite solar cells.

Observing the optimization history scatter plots (subfigure (h) in Figure 4, Figure 5, Figure 6, Figure 7, Figure 8, Figure 9 and Figure 10) of each algorithm, it is evident that PSO and GWO rapidly approached the high-efficiency region in early iterations with a broad search scope, achieving convergence with a lower total number of evaluations. This indicates that such swarm intelligence algorithms possess extremely high search efficiency and the ability to escape local optima when processing high-dimensional spaces. Although the SA ultimately achieved the same accuracy, its high-temperature characteristics in the early stages led to extensive random exploration within low-PCE regions, relying on a larger number of iteration steps to finally cool down and converge. Both BO and Differential Evolution failed to reach the best observed limit, falling into local optima under the current stopping criteria. For instance, as seen in Figure 10g, the BO algorithm extensively sampled near 3 × 10^13^ when optimizing the acceptor doping concentration of RbGeI_3_, whereas the best optimization algorithms yielded a result of 1 × 10^13^. Notably, all trial points achieving high PCE were highly concentrated in the physical boundary regions of specific parameters, such as the extremely low defect density range. This optimization behavior highly consistently captured the decisive hindering effect of interface recombination on the performance of double-absorber devices, verifying the reliability of the surrogate model and optimization algorithms at the physical level.

**Figure 11:**
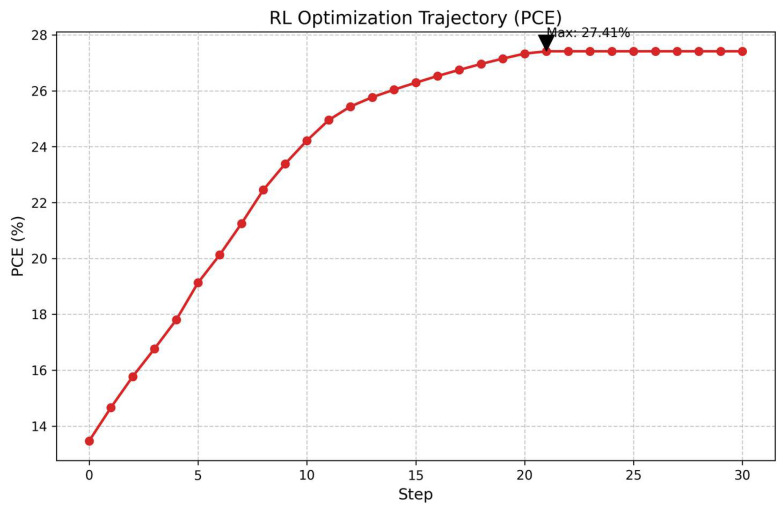
Optimization history of the RL agent.

The RL agent demonstrates a starkly different optimization paradigm within the optimization framework of this study. Figure 11 illustrates the PCE optimization history of the RL agent starting from a random initial point for 30 steps. Unlike the historical trajectories of traditional heuristic algorithms, which are scattered with random trial points, the RL agent, after undergoing 100,000 pre-training iterations, internalizes the implicit nonlinear mapping relationships between the 7 physical parameters and the PCE into a policy network. From the RL optimization trajectory plot, it can be observed that during the testing phase, the agent exhibits a smooth and monotonically increasing optimization curve. Through 21 decision steps, the agent deterministically converges to the highest PCE of 27.41% via precise, continuous action fine-tuning.

The results show that in the optimization based on the surrogate model, SA, PSO, GA, GWO, and RL all achieved the same maximum PCE (27.41%), corresponding to a relative deviation of 0% from the best result. While the simplex method NM, DE, and Bayesian optimization BO had relative deviations of 0.51%, 0.62%, and 1.42%, respectively, from the best result. Therefore, for the established goals of high-throughput device screening, the final PCE differences among most of the algorithms are very small.

#### 3.2.2. Evaluation of Algorithm Optimization Efficiency and Accuracy Based on the SCAPS-1D Automated Framework

For novel device architectures lacking sufficient data to train surrogate models, a more comprehensive optimization of device parameters typically requires consideration of a larger number of variables. In this study, the developed automated optimization framework is utilized to systematically optimize 15-dimensional variables, including the thicknesses, doping concentrations, bulk defect densities, and interface defect densities of the absorber layers and charge transport layers, as well as the work function of the back electrode. Among these, the first seven dimensional variables and their value ranges are consistent with Table 2. All optimized variables and their corresponding ranges are presented in Table 5. Each optimization algorithm undergoes 15 independent tests to mitigate the influence of randomness on the experimental results, and a random search is introduced to demonstrate the high efficiency of incorporating optimization algorithms into real device simulation optimization. For NM, PSO, GA, DE, GWO, BO, and the random search, the number of function evaluations (NFE) for each test is set to 400 to fairly compare the optimization efficiency of different algorithms. For SA, optimization automatically terminates when the temperature reaches 1 × 10^−9^.

**Figure 12:**
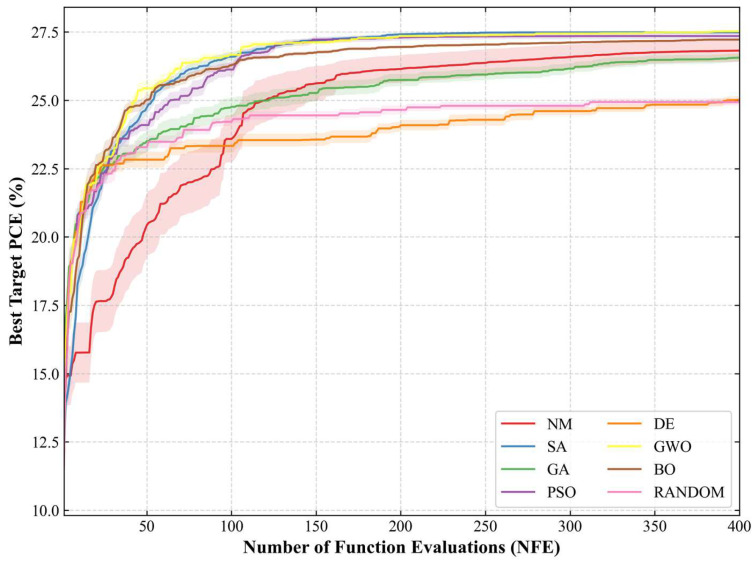
Average convergence curves of PCE optimized by different optimization algorithms over 15 independent runs. The solid lines represent the average best-so-far PCE, and the shaded regions represent 20% of the corresponding standard deviation.

The optimization performance of the different algorithms is illustrated in Figure 12, where the solid lines represent the relationship between the average maximum PCE achieved by the corresponding optimization algorithm and the NFE. To prevent overlapping in the figure, the error bands indicated by the shaded areas are uniformly set to 20% of the standard deviation. The final optimized PCE data and standard deviations for each algorithm are provided in Supplementary Information Table S1. It can be observed from the figure that the optimization results of all algorithms surpassed those of the random search. GWO, BO, and SA require only about 50 evaluations to exceed the best result obtained from 400 random searches, demonstrating the effectiveness of the optimization algorithms. In the early stage of optimization (within 100 evaluations), BO and GWO exhibited higher optimization efficiency, indicating stronger early convergence capabilities. As the number of optimization iterations increased, GWO, PSO, and SA demonstrated superior optimization performance. Ultimately, the optimized results reach 27.52 ± 0.20% for GWO, 27.48 ± 0.14% for SA, and 27.35 ± 0.55% for PSO. For DE and GA, the results indicate that 400 physical simulations were insufficient for them to optimize to the device’s optimal level, rendering them unsuitable for physical simulation optimization using the automated framework.

Table 6 further quantifies the computational overhead required for each algorithm to reach the target efficiency, which was set as 98% of the optimal PCE of 27.73%, i.e., 27.18%. To further evaluate the percentage difference in optimization efficiency, the reduction in physical simulation cost was calculated using Equation (12).

(12) $$R_{i}=\frac{N-{\bar{NFE}}_{i}}{N}\times 100\%$$

N is the maximum number of SCAPS-1D evaluations, which was set to 400 for most algorithms in this study, and ${\bar{NFE}}_{i}$ is the average number of function evaluations required by algorithm i to reach the target PCE among successful runs. The results show that SA and PSO reached the target region most efficiently, requiring only 138.2 ± 46.9 and 110.7 ± 31.6 physical evaluations on average, respectively. Relative to the maximum budget of 400 SCAPS-1D evaluations, this corresponds to reductions of approximately 65.5% and 72.3%, respectively. GWO also showed high efficiency, reaching the target PCE in 160.9 ± 116.9 evaluations on average, corresponding to a 59.8% reduction in physical simulation cost. In comparison, NM, BO, and GA required 226.5, 230.2, and 271.3 evaluations on average among successful runs, corresponding to smaller reductions of 43.4%, 42.5%, and 32.2%, respectively. Moreover, their success rates were lower than those of SA, PSO, and GWO, especially for GA, which reached the target in only 3 out of 15 independent runs. DE and random search failed to reach the target PCE within the given evaluation budget. These results indicate that, for the intended goal of reducing the computational cost of direct SCAPS-1D-based optimization, the percentage differences in optimization efficiency were substantial. Although several algorithms could eventually approach a high-efficiency region, SA and PSO provided the most favorable balance between convergence speed, success rate, and simulation cost.

**Figure 13:**
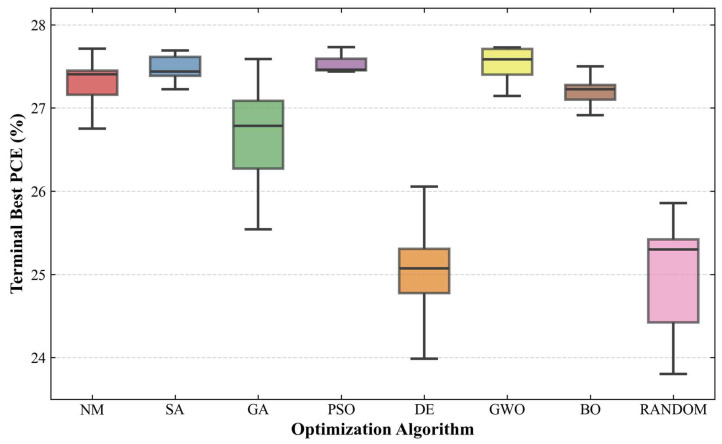
Box plots of the best PCE achieved by different optimization algorithms across 15 independent optimizations.

Beyond convergence efficiency, an algorithm’s optimization robustness within a complex physical space is equally a core metric of concern in device design. The box plots in Figure 13 visually compare the distributions of the highest PCEs obtained by each algorithm under 15 independent initializations. The results show that while GWO possesses the highest median among all algorithms, its large degree of dispersion indicates poor robustness. This is further evidenced by Table 6, where the standard deviation of GWO in achieving 98% of the optimal PCE is the highest among all algorithms, reaching 116.9. SA ranked second in median among all algorithms, accompanied by a smaller degree of dispersion. PSO and BO exhibit narrower boxes in Figure 13 and smaller standard deviations for achieving 98% of the optimal PCE, demonstrating outstanding stability that is highly insensitive to initial states. Conversely, none of the DE algorithm trials reached the target benchmark in this test. Similar to the GA, its optimization result distribution exhibits severe variance fluctuation. Combined with the analysis in Section 3.2.1, this is attributed to the algorithm’s inability to converge rapidly within merely 400 physical simulations.

### 3.3. Generalization Performance of Transfer Learning Across Structures

**Figure 14:**
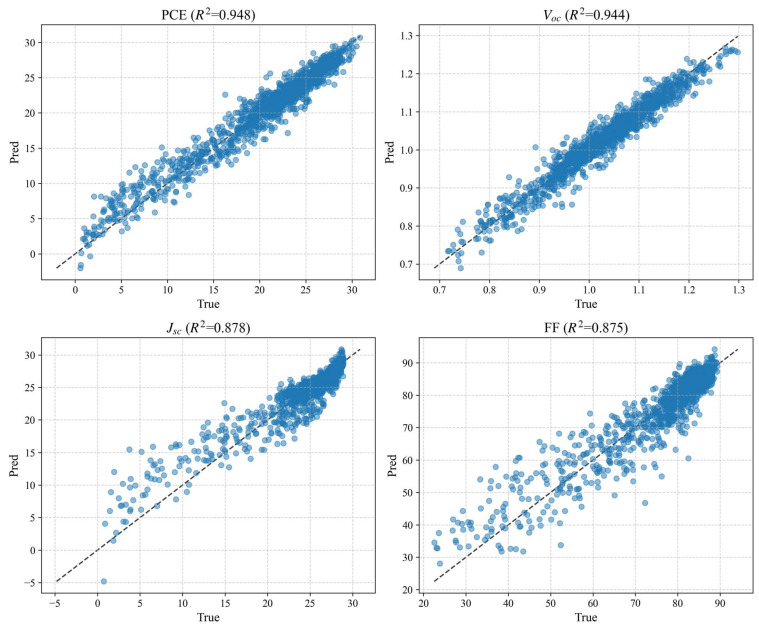
Performance of the model trained directly using target domain data.

**Figure 15:**
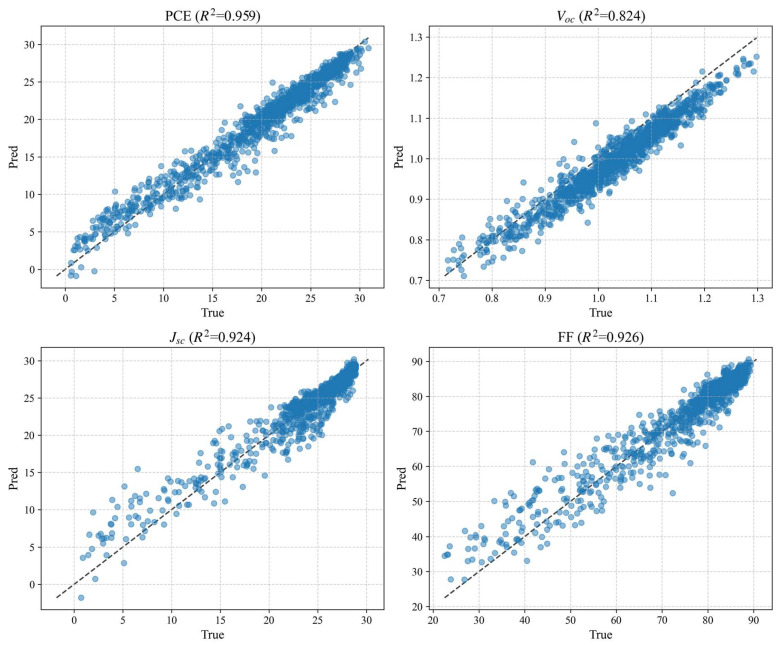
Performance of the model trained using transfer learning.

For the FASnI_3_/Sb_2_S_3_ structure, the prediction performance of the MLP model trained directly on 225 sets of simulation data, evaluated on an independent test set of 1275 sets, is shown in Figure 14. The test set prediction performance of the model fine-tuned from the pre-trained model developed in Section 3.1 using the same data is shown in Figure 15. To provide a physically meaningful evaluation, Table 7 summarizes the output-specific results for the four photovoltaic parameters using R^2^, RMSE, MAE, and NRMSE.

For PCE, transfer learning increases R^2^ from 0.948 to 0.959 and reduces the RMSE from 1.571% to 1.399%, while the NRMSE decreases from 5.18% to 4.62%. For J_SC_, R^2^ increases from 0.878 to 0.924, and the RMSE decreases from 1.800 mA/cm^2^ to 1.419 mA/cm^2^. Similarly, for FF, R^2^ increases from 0.875 to 0.926, and the RMSE decreases from 4.726% to 3.638%. These results indicate that transfer learning improves the prediction of PCE, J_SC_, and FF under limited target-domain data.

For the V_OC_ parameter, the predicted values after transfer learning are generally underestimated. This is because, without considering recombination losses and band bending, the theoretical open-circuit voltage of the FASnI_3_/Sb_2_S_3_ solar cell structure is 1.3 V, whereas that of the Cs_2_TiBr_6_/RbGeI_3_ solar cell structure is 1.21 V. The relatively large difference in the numerical mapping between the source and target domains leads to a partial negative-transfer effect for V_OC_. These findings indicate that transfer learning can reduce the demand for target-domain data and improve the cross-structure prediction capability for several key photovoltaic parameters. However, output-specific evaluation remains essential for identifying possible negative transfer in individual performance metrics. In future explorations of unknown photovoltaic materials and structures, the method of applying transfer learning based on existing device modeling can substantially reduce data acquisition costs and rapidly assist in evaluating the performance of new device structures.

A full list of abbreviations used in this study is provided in Supplementary Information Table S2.

## 4. Conclusions

This study presents an end-to-end optimization framework for lead-free double-absorber Cs_2_TiBr_6_/RbGeI_3_ perovskite solar cells by integrating automated physical simulation, high-accuracy surrogate modeling, multidimensional intelligent optimization, and transfer learning. The results show that the MLP surrogate model, trained on a limited dataset generated by Latin Hypercube Sampling, achieves high predictive accuracy (R^2^ = 0.97) and effectively replaces time-consuming SCAPS-1D simulations. Based on this surrogate model, the PPO-based reinforcement learning agent learns the nonlinear relationship between seven physical parameters and PCE, and converges smoothly to an optimal PCE of 27.41% within only a few decision steps, while SA, PSO, GA, and GWO also demonstrate strong optimization performance in the surrogate-model environment. In direct 15-dimensional optimization using the automated SCAPS-1D framework, SA shows the best balance between convergence accuracy and search efficiency, requiring only 138.2 physical evaluations on average to approach the target optimum, whereas GWO achieves the highest average PCE of 27.52% but with lower robustness. In addition, transfer learning further improved model generalization across device structures: after fine-tuning the pretrained Cs_2_TiBr_6_/RbGeI_3_ model on the FASnI_3_/Sb_2_S_3_ target domain using only 225 samples, the resulting model outperforms direct training, with improved R^2^ values for PCE, J_SC_, and FF and a reduced overall test-set RMSE. These results demonstrate the effectiveness of combining surrogate modeling, intelligent optimization, and transfer learning for efficient photovoltaic device design under both high-dimensional and data-scarce conditions.

## Figures and Tables

**Table 1:** Input parameters used in SCAPS-1D simulations.

Material	FTO	ZnO	Cs_2_TiBr_6_	RbGeI_3_	CuI
**Thickness (μm)**	0.1	0.1	0.3	0.3	0.1
**Band gap (eV)**	3.6	3.3	1.8	1.31	3.1
**Electron affinity (eV)**	4	4	4	3.9	2.1
**Relative dielectric permittivity**	9	9	10	23.01	6.5
**Conduction band minimum (eV, vs. vacuum)**	−4	−4	−4	−3.9	−2.1
**Valence band maximum (eV, vs. vacuum)**	−7.6	−7.3	−5.8	−5.21	−5.2
**CB effective DOS (cm^−3^)**	2.2 × 10^18^	2.2 × 10^18^	6 × 10^19^	1.8 × 10^18^	2.8 × 10^19^
**VB effective DOS (cm^−3^)**	1.8 × 10^19^	1.8 × 10^19^	2.14 × 10^19^	1 × 10^18^	1.0 × 10^19^
**Electron mobility (cm^2^/Vs)**	100	100	0.236	28.6	100
**Hole mobility (cm^2^/Vs)**	25	25	0.171	27.3	43.9
**Donor density ND (cm^−3^)**	1 × 10^18^	1 × 10^18^	1 × 10^13^	0	0
**Acceptor density NA (cm^−3^)**	0	0	0	1 × 10^13^	1 × 10^18^
**Defect type**	Neutral	Neutral	Neutral	Neutral	Neutral
**Defect density N_t_ (cm^−3^)**	1 × 10^14^	1 × 10^15^	1 × 10^15^	1 × 10^15^	1 × 10^15^
**References**	[37]	[38]	[39]	[40]	[41]

**Table 2:** Sampling ranges of input parameters.

Parameters	Data Range
Cs_2_TiBr_6_ thickness (μm)	0.15~0.8
RbGeI_3_ thickness (μm)	0.15~0.8
Cs_2_TiBr_6_ defect density (cm^−3^)	10^13^~10^18^
RbGeI_3_ defect density (cm^−3^)	10^13^~10^18^
Cs_2_TiBr_6_/RbGeI_3_ interface defect density (cm^−2^)	10^13^~10^15^
Cs_2_TiBr_6_ donor density (cm^−3^)	10^13^~10^17^
RbGeI_3_ acceptor density (cm^−3^)	10^13^~10^17^

**Table 3:** Original dataset.

Type	Parameters	Data Number				
		1	2	3	…	1500
Independent variable	Cs_2_TiBr_6_ thickness (μm)	0.424	0.210	0.439	…	0.463
	RbGeI_3_ thickness (μm)	0.431	0.439	0.576	…	0.799
	Cs_2_TiBr_6_ defect density (cm^−3^)	6.314 × 10^16^	3.273 × 10^15^	3.926 × 10^15^	…	9.590 × 10^14^
	RbGeI_3_ defect density (cm^−3^)	1.432 × 10^13^	1.006 × 10^13^	9.622 × 10^15^	…	1.121 × 10^14^
	Cs_2_TiBr_6_/RbGeI_3_ interface defect density (cm^−2^)	7.642 × 10^14^	5.263 × 10^13^	8.253 × 10^13^	…	1.481 × 10^13^
	Cs_2_TiBr_6_ donor density (cm^−3^)	9.269 × 10^16^	3.788 × 10^14^	6.569 × 10^15^	…	1.813 × 10^15^
	RbGeI_3_ acceptor density (cm^−3^)	9.361 × 10^15^	5.535 × 10^15^	5.465 × 10^16^	…	3.804 × 10^14^
Dependent variable	PCE (%)	10.17	19.20	14.02	…	17.67
	V_OC_ (V)	0.76	0.83	0.84	…	0.89
	J_SC_ (mA/cm^2^)	16.51	31.35	27.69	…	31.94
	FF (%)	81.51	73.88	60.53	…	62.43

**Table 4:** Optimization results of different methods on the surrogate model.

Methods	NM	SA	PSO	GA	DE	GWO	BO	RL
NFE	1400	2225	801	4638	3490	744	2509	100,000
Max PCE (%)	27.27	27.41	27.41	27.41	27.24	27.41	27.02	27.41

**Table 5:** Input parameters and their value ranges during optimization using the SCAPS-1D automated framework.

Input Parameters	Data Range
Cs_2_TiBr_6_ thickness (μm)	0.15~0.75
RbGeI_3_ thickness (μm)	0.15~0.75
Cs_2_TiBr_6_ defect density (cm^−3^)	10^13^~10^18^
RbGeI_3_ defect density (cm^−3^)	10^13^~10^18^
Cs_2_TiBr_6_/RbGeI_3_ interface defect density (cm^−2^)	10^13^~10^15^
Cs_2_TiBr_6_ donor density (cm^−3^)	10^13^~10^17^
RbGeI_3_ acceptor density (cm^−3^)	10^13^~10^17^
ZnO thickness (μm)	0.03~0.17
CuI thickness (μm)	0.03~0.17
ZnO donor density (cm^−3^)	10^15^~10^20^
CuI acceptor density (cm^−3^)	10^15^~10^20^
RbGeI_3_/CuI interface defect density (cm^−2^)	10^13^~10^15^
ZnO/Cs_2_TiBr_6_ interface defect density (cm^−2^)	10^13^~10^15^
FTO/ZnO interface defect density (cm^−2^)	10^13^~10^15^
Back contact work function (eV)	4.8~5.2

**Table 6:** Average NFE required for different optimization algorithms to reach 98% of the optimal efficiency.

Algorithm	Target PCE (%)	Success Rate	Avg NFE (Successful Runs)	NFE Std
NM	27.1754	11/15	226.5	88.7
SA		15/15	138.2	46.9
GA		3/15	271.3	95.5
PSO		13/15	110.7	31.6
DE		0/15	-	-
GWO		14/15	160.9	116.9
BO		9/15	230.2	80.6
RANDOM		0/15	-	-

**Table 7:** Output-specific prediction metrics of direct training and transfer learning on the target-domain independent test set.

Model	Output	R^2^	RMSE	MAE	NRMSE (%)
Direct training	PCE (%)	0.948	1.571	1.196	5.18
Direct training	V_OC_ (V)	0.944	0.0247	0.0189	4.24
Direct training	J_SC_ (mA/cm^2^)	0.878	1.8	1.138	6.38
Direct training	FF (%)	0.875	4.726	3.236	7.07
Transfer learning	PCE (%)	0.959	1.399	1.014	4.62
Transfer learning	V_OC_ (V)	0.824	0.0435	0.0382	7.49
Transfer learning	J_SC_ (mA/cm^2^)	0.924	1.419	0.877	5.03
Transfer learning	FF (%)	0.926	3.638	2.365	5.44

## Data Availability

The original contributions presented in this study are included in the article/Supplementary Materials. Further inquiries can be directed to the corresponding author.

## Supplementary Materials

The following supporting information can be downloaded at: https://www.mdpi.com/article/10.3390/ma19143091/s1. Figure S1: Energy level diagram of the electron transport layer and the absorption layer; Figure S2: Energy level diagram of the hole transport layer and the absorption layer; Table S1: The mean and standard deviation of PCE optimized by different optimization algorithms; Table S2: List of abbreviations used in the manuscript; Video S1: automated optimization process.
