# Supplementary material for: Accelerating the Design of Double-Absorber Solar Cells: From Surrogate Model-Assisted Reinforcement Learning and Multi-Algorithm Optimization Comparison to Transfer Learning
Source: Materials (Basel). 2026 Jul 17;19(14):3091. doi: 10.3390/ma19143091 (PMC13414044; doi:10.3390/ma19143091)
Supplement: Supplementary file 1 [file materials-19-03091-s001.zip › Supplementary Information.pdf]

Supporting Information

Accelerating the Design of Double-absorber Solar Cells: From Surrogate Model-Assisted Reinforcement Learning and Multi-Algorithm Optimization Comparison to Transfer Learning

Yuhan Zhang, Qiaochu Sun, Jiang Zhao*

College of Integrated Circuit Science and Engineering, Nanjing University of Posts and Telecommunications, Nanjing 210023, P. R. China

*Corresponding author, E-mail: jzhao@njupt.edu.cn (J. Zhao)

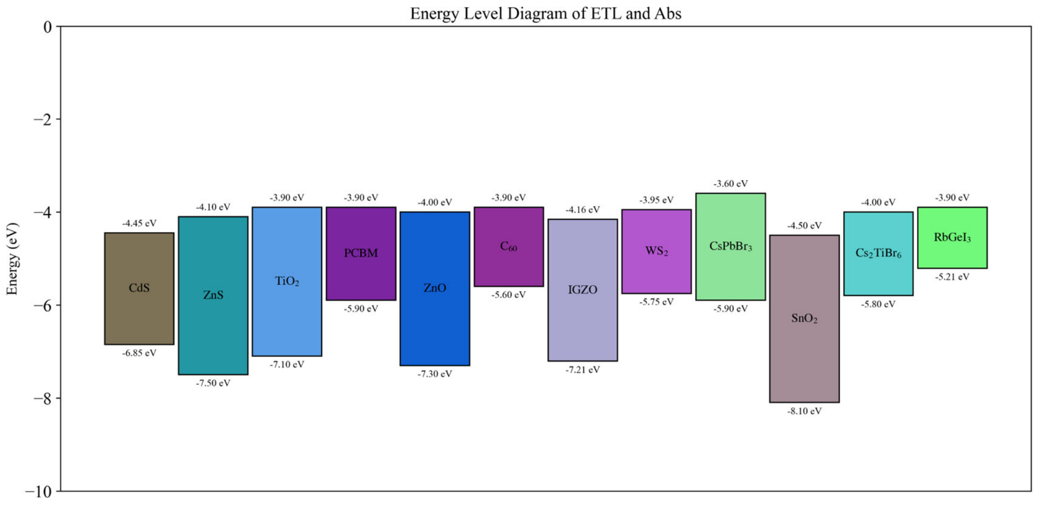

Figure S1. Energy level diagram of the electron transport layer and the absorption layer.

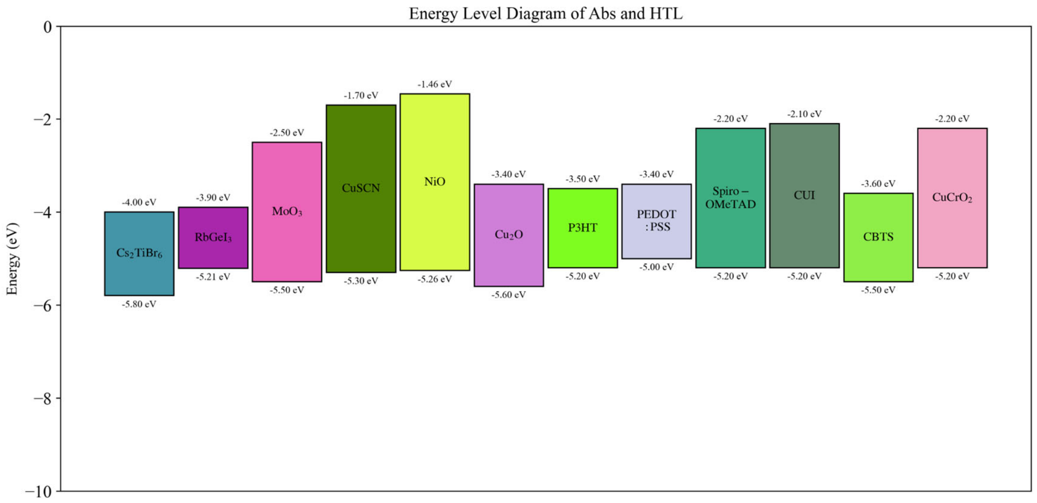

Figure S2. Energy level diagram of the hole transport layer and the absorption layer.

Table S1. The mean and standard deviation of PCE optimized by different optimization algorithms

Algorithms	**Average Maximum PCE**	**Standard deviation of the average maximum PCE**	**Average standard deviation**
GWO	27.5202	0.2005	0.6252
SA	27.4827	0.143	0.5485
PSO	27.3487	0.5513	0.8535
BO	27.2176	0.1885	0.5391
NM	26.8146	2.0335	3.3218
GA	26.5555	0.7582	1.338
DE	25.0153	0.6603	1.1088
RANDOM	24.9322	0.6559	0.8835

Table S2. List of abbreviations used in the manuscript.

**Abbreviation**	**Full term**
ANN	Artificial Neural Network
BO	Bayesian Optimization
CBO	Conduction Band Offset
DE	Differential Evolution
DFT	Density Functional Theory
DOS	Density of States
ETL	Electron Transport Layer
FTO	Fluorine-Doped Tin Oxide
GA	Genetic Algorithm
GWO	Grey Wolf Optimizer
HTL	Hole Transport Layer
LHS	Latin Hypercube Sampling
MAE	Mean Absolute Error
ML	Machine Learning
MLP	Multilayer Perceptron
MSE	Mean Squared Error
NFE	Number of Function Evaluations
NM	Nelder-Mead method

Abbreviation	Full term
NRMSE	Normalized Root Mean Square Error
PPO	Proximal Policy Optimization
PSO	Particle Swarm Optimization
R^2	Coefficient of Determination
ReLU	Rectified Linear Unit
RF	Random Forest
RL	Reinforcement Learning
RMSE	Root Mean Square Error
SA	Simulated Annealing
SHAP	SHapley Additive exPlanations
SVR	Support Vector Regression
VBO	Valence Band Offset
XGBoost	eXtreme Gradient Boosting
